# Standardized pipelines support and facilitate integration of diverse datasets at the Rat Genome Database

**DOI:** 10.1093/database/baae132

**Published:** 2025-01-22

**Authors:** Jennifer R Smith, Marek A Tutaj, Jyothi Thota, Logan Lamers, Adam C Gibson, Akhilanand Kundurthi, Varun Reddy Gollapally, Kent C Brodie, Stacy Zacher, Stanley J F Laulederkind, G Thomas Hayman, Shur-Jen Wang, Monika Tutaj, Mary L Kaldunski, Mahima Vedi, Wendy M Demos, Jeffrey L De Pons, Melinda R Dwinell, Anne E Kwitek

**Affiliations:** Rat Genome Database, Department of Physiology, Medical College of Wisconsin, 8701 Watertown Plank Rd, Milwaukee, WI 53226, United States; Rat Genome Database, Department of Physiology, Medical College of Wisconsin, 8701 Watertown Plank Rd, Milwaukee, WI 53226, United States; Rat Genome Database, Department of Physiology, Medical College of Wisconsin, 8701 Watertown Plank Rd, Milwaukee, WI 53226, United States; Rat Genome Database, Department of Physiology, Medical College of Wisconsin, 8701 Watertown Plank Rd, Milwaukee, WI 53226, United States; Rat Genome Database, Department of Physiology, Medical College of Wisconsin, 8701 Watertown Plank Rd, Milwaukee, WI 53226, United States; Rat Genome Database, Department of Physiology, Medical College of Wisconsin, 8701 Watertown Plank Rd, Milwaukee, WI 53226, United States; Rat Genome Database, Department of Physiology, Medical College of Wisconsin, 8701 Watertown Plank Rd, Milwaukee, WI 53226, United States; Clinical and Translational Science Institute, Medical College of Wisconsin, 8701 Watertown Plank Rd, Milwaukee, WI 53226, United States; Finance and Administration, Medical College of Wisconsin, 8701 Watertown Plank Rd, Milwaukee, WI 53226, United States; Rat Genome Database, Department of Physiology, Medical College of Wisconsin, 8701 Watertown Plank Rd, Milwaukee, WI 53226, United States; Rat Genome Database, Department of Physiology, Medical College of Wisconsin, 8701 Watertown Plank Rd, Milwaukee, WI 53226, United States; Rat Genome Database, Department of Physiology, Medical College of Wisconsin, 8701 Watertown Plank Rd, Milwaukee, WI 53226, United States; Rat Genome Database, Department of Physiology, Medical College of Wisconsin, 8701 Watertown Plank Rd, Milwaukee, WI 53226, United States; Rat Genome Database, Department of Physiology, Medical College of Wisconsin, 8701 Watertown Plank Rd, Milwaukee, WI 53226, United States; Rat Genome Database, Department of Physiology, Medical College of Wisconsin, 8701 Watertown Plank Rd, Milwaukee, WI 53226, United States; Rat Genome Database, Department of Physiology, Medical College of Wisconsin, 8701 Watertown Plank Rd, Milwaukee, WI 53226, United States; Rat Genome Database, Department of Physiology, Medical College of Wisconsin, 8701 Watertown Plank Rd, Milwaukee, WI 53226, United States; Rat Genome Database, Department of Physiology, Medical College of Wisconsin, 8701 Watertown Plank Rd, Milwaukee, WI 53226, United States; Rat Genome Database, Department of Physiology, Medical College of Wisconsin, 8701 Watertown Plank Rd, Milwaukee, WI 53226, United States

## Abstract

The Rat Genome Database (RGD) is a multispecies knowledgebase which integrates genetic, multiomic, phenotypic, and disease data across 10 mammalian species. To support cross-species, multiomics studies and to enhance and expand on data manually extracted from the biomedical literature by the RGD team of expert curators, RGD imports and integrates data from multiple sources. These include major databases and a substantial number of domain-specific resources, as well as direct submissions by individual researchers. The incorporation of these diverse datatypes is handled by a growing list of automated import, export, data processing, and quality control pipelines. This article outlines the development over time of a standardized infrastructure for automated RGD pipelines with a summary of key design decisions and a focus on lessons learned.

## Introduction

The Rat Genome Database (RGD, https://rgd.mcw.edu [1]) was originally developed as an online resource for data for the laboratory rat, *Rattus norvegicus*, and as a comparative data platform for rat, mouse, and human [2, 3]. Since its inception in 1999, RGD has expanded into a multispecies knowledgebase with integrated genetic, multiomic, phenotypic, and disease data across 10 mammalian species, including rat, human, mouse, dog, pig, chinchilla, 13-lined ground squirrel, bonobo, green monkey, and naked mole-rat [4], and a suite of species-agnostic tools for finding, retrieving, visualizing, exploring, and utilizing those data [5].

## Data integration, standardization, and harmonization

RGD integrates data derived from expert manual curation by the team of RGD research scientists and biocurators with both informatically generated and manually curated data imported from other databases, as well as direct data submissions from the research community. This integration of data from multiple sources provides researchers with a broader understanding of what is known about, for instance, a gene or rat strain. RGD allows researchers to view and utilize these data in a single location, while maintaining the provenance and linking back to the original sources, whether those are research articles or source databases, for researchers who need more details.

To give an indication of the extensive data and metadata integration that RGD provides, Table 1 contains a list of sources [6–46] from which RGD imports data objects, metadata such as external database links, and ontology annotations. These imports complement and extend the data that RGD curators manually extract from the literature. Integration of these data facilitates their use and, in the case of ontology annotations, allows RGD to propagate the information to orthologous genes in other, less well-studied species where appropriate. For emerging models such as chinchilla and squirrel, this provides a sizable corpus of disease, pathway, and Gene Ontology (GO) annotations derived from the manual curation of literature for human and other more extensively studied and curated models. Although the based-on-orthology annotations are not themselves experimentally supported in the species receiving these annotations, they can provide valuable pointers for researchers attempting to ascertain the genetic basis of complex diseases in their models.

**Table 1.** Sources of data for RGD’s import pipelines

Data object and metadata source databases	Data retrieved	Species directly affected	Database URL	Database reference(s)
Alliance of Genome Resources	Gene descriptions, ortholog assignments, BioGRID protein–protein interactions	Rat, human, mouse	https://www.alliancegenome.org/	Alliance of Genome Resources Consortium 2022; Alliance of Genome Resources Consortium 2024
AlphaFold	Protein 3D structures	Rat, human, mouse	https://alphafold.ebi.ac.uk	Varadi *et al*. 2024
Cellosaurus	Cell line records	All except chinchilla	https://web.expasy.org/cellosaurus/	Bairoch 2018
ClinVar	Clinical variants, disease and human phenotype (HPO) annotations for variants and genes	Human	https://www.ncbi.nlm.nih.gov/clinvar/	Landrum *et al*. 2020; Sayers *et al*. 2024
Comparative Toxicogenomics Database (CTD)	Gene–chemical interaction annotations, DO annotations	Rat, human, mouse, dog, pig, green monkey	http://ctdbase.org/	Davis *et al*. 2021
COSMIC	External database IDs to COSMIC database	Human	https://cancer.sanger.ac.uk/cosmic	Sondka *et al*. 2024
dbSNP	Genomic variants	Human	https://www.ncbi.nlm.nih.gov/snp/	Sayers *et al*. 2024
EMBL-EBI GOA Database	GOAs	All except mouse	https://www.ebi.ac.uk/GOA/index	Huntley *et al*. 2015
Ensembl	Gene records, xrefs	All	https://www.ensembl.org/index.html	Harrison *et al*. 2024
Eukaryotic Promoter Database (EPD)	Gene promoters	Rat, human, mouse, dog	https://epd.epfl.ch//index.php	Meylan *et al*. 2020
EVA	Genomic variants	Rat, mouse, dog, pig, green monkey	https://www.ebi.ac.uk/eva/	Cezard *et al*. 2022
Expression Atlas	RNA-Seq-based gene expression metadata and values	Rat, human	https://www.ebi.ac.uk/gxa/home	George *et al*. 2024
Gene Expression Omnibus (GEO)	RNA-Seq-based gene expression metadata and values	Rat, human	https://www.ncbi.nlm.nih.gov/geo/	Clough *et al*. 2024
GOC	GOAs	Mouse	http://geneontology.org/	Ashburner *et al*. 2000; Aleksander *et al*. 2023
GTEx	RNA-Seq-based gene expression values	Human	https://gtexportal.org/home/	GTEx Consortium 2020
HGNC Comparison of Orthology Predictions (HCOP)	Ortholog assignments	Rat, human, mouse, dog, pig	https://www.genenames.org/tools/hcop/	Yates *et al*. 2021
HUGO Gene Nomenclature Committee (HGNC) Database	Gene nomenclature	Human	https://www.genenames.org/	Seal *et al*. 2023
HPO group	HPO annotations	Human	https://hpo.jax.org/app/	Gargano *et al*. 2024
IMEx	Protein–protein interactions	Rat, human, mouse, dog, pig	https://www.imexconsortium.org/	Porras *et al*. 2020
miRBase	Verified and predicted miRNA targets	Rat, human, mouse	https://www.mirbase.org/	Kozomara *et al*. 2019
Mouse Genome Informatics (MGI)	quantitative trait loci (QTLs), gene and QTL mammalian phenotype (MP) annotations, gene nomenclature	mouse	http://www.informatics.jax.org/	Ringwald *et al*. 2022; Bult *et al*. 2019
NCBI	Gene records, transcript records, transcript sequences, protein sequences, ortholog assignments, xrefs	All	https://www.ncbi.nlm.nih.gov/	Sayers *et al*. 2024
NHGRI-EBI GWAS Catalog	Genome-wide association study variants	Human	https://www.ebi.ac.uk/gwas/	Sollis *et al*. 2023
Online Mendelian Inheritance in Animals (OMIA)	DO annotations	Dog, pig	https://www.omia.org/home/	Nicholas *et al*. 2021
OMIM	DO annotations, external database IDs for OMIM disease and gene records	Human	https://www.omim.org/	Amberger *et al*. 2019
Rat GTEx project, NIDA Center for Genetic Studies of Drug Abuse in Outbred Rats (P50DA037844)	External database IDs to the RatGTEx Portal	Rat	https://ratgtex.org/	Munro *et al*. 2022
RNACentral	Xrefs for noncoding gene records	Rat, human, mouse, bonobo, dog, pig, green monkey	https://rnacentral.org/	RNAcentral Consortium 2021
UCSC Genome Browser Database	Synteny data	Rat, human, mouse, bonobo, dog, pig, green monkey	https://genome.ucsc.edu	Raney *et al*. 2024
UniProtKB	Xrefs, protein sequences, canonical proteins, protein domains	All	https://www.uniprot.org/help/uniprotkb	UniProt Consortium 2023
Vertebrate Gene Nomenclature Committee Database	Gene nomenclature	Dog, pig	https://vertebrate.genenames.org/	Jones *et al*. 2023
**Ontology file sources**	**Data retrieved**	**Species directly affected**	**Source URL**	**Ontology and database reference(s)**
DO Knowledgebase	DO files	n/a	https://disease-ontology.org/	Baron *et al*. 2024
European Bioinformatics Institute (EBI)	Chemical entities of biological interest ontology files	n/a	https://ftp.ebi.ac.uk/pub/databases/chebi/ontology/	Hastings *et al*. 2016
GOC	Gene ontology files	n/a	http://geneontology.org/	Ashburner *et al*. 2000; Aleksander *et al*. 2023
GitHub/obophenotype	Cell ontology and Uberon multispecies anatomy ontology files	n/a	https://github.com/obophenotype	Diehl *et al*. 2016 (CL); Haendel *et al*. 2014 (UBERON)
GitHub/Sequence Ontology	Sequence types and features ontology files	n/a	https://github.com/The-Sequence-Ontology/	Mungall *et al*. 2011
MGI	Mammalian phenotype and mouse adult gross anatomy ontology files	n/a	http://www.informatics.jax.org/	Ringwald *et al*. 2022; Bult *et al*. 2019
OBO Foundry	HPO and vertebrate trait ontology files	n/a	http://obofoundry.org/	Jackson *et al*. 2021 (OBO Foundry); Kohler *et al*. 2021 (HPO); Park *et al*. 2013 (VT)
Swiss Institute of Bioinformatics Expasy	Cellosaurus vocabulary files	n/a	https://www.expasy.org/	Duvaud *et al*. 2021 (Expasy); Bairoch 2018 (Cellosaurus)

List of resources from which RGD imports data, with information about what data are imported, which RGD species are directly associated with the imported data, the URL for each resource, and one or more references for each resource.

The use of automated pipeline software to perform the integration of external data ensures that the data are incorporated on a regular basis and therefore remain up to date. Relatively little ongoing effort is necessary from RGD developers and curators beyond updates required in response to changes in the incoming data files and improvements to enhance the functionality of a given pipeline. To facilitate the addition of new pipelines, RGD has created a standardized infrastructure upon which software can be easily built. As will be outlined later, the reuse of the basic pipeline code facilitates and accelerates development and allows creation and maintenance of RGD pipelines to be distributed across the RGD development team. The decision to utilize an incremental update strategy for pipelines will be discussed, as will key aspects of the pipelines’ functionality such as the handling of nonharmonized data from diverse sources.

## The drop-and-reload paradigm

Extract–transform–load (ETL) pipelines can follow either a drop-and-reload or an incremental update paradigm [47]. A fully drop-and-reload process begins with an empty datastore and loads data from incoming data sources via one or more ETL pipelines. At each step, the data loading process can be, and often is, dependent on data loaded by other pipelines, making the entire process sequential. With each pipeline run, the entire target dataset is built from scratch from the incoming data.

The drop-and-reload paradigm has the advantages that the concept is straightforward and easy to understand, and the initial implementation is relatively simple. For these reasons, this process is often utilized as the starting point for a project. Drop-and-reload works well, particularly for smaller datasets with well-defined incoming data where loads will be fast and relatively few problems are anticipated.

The initial data imports implemented at the Alliance of Genome Resources (the Alliance) [7, 8], of which RGD is a founding member, represent an example of the successful use of the drop-and-reload paradigm. Started in 2016, the Alliance was created to provide a shared infrastructure and a unified look and feel across originally six, now seven, model organism databases (MODs) [38, 48–52] and the Gene Ontology Consortium (GOC) [6, 10]. From the early days of the Alliance, a substantial amount of work has been done to harmonize the representation of shared datatypes such as gene records, disease annotations, and protein–protein interactions. In the same way that the GOC has created a standardized format for sharing GO annotations, the Alliance has worked to create a shared species-agnostic data model for MODs to submit data. Because the incoming data were all submitted using a single shared JSON schema for each data type, they could be standardized at the source. In addition, each MOD acted as the “source of truth” for the data it provided—changes that needed to be made were made at the MOD, not at the Alliance—so dropping the existing data from the Alliance database and replacing them with the data coming from the truth source was the most logical option and was implemented successfully.

**Figure 1.**
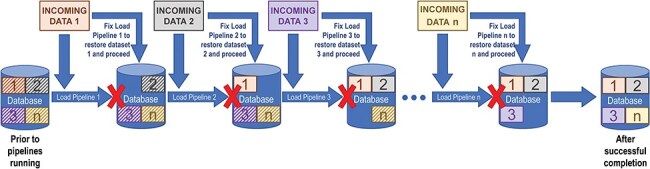
The drop-and-reload paradigm. Dropping and reloading data introduces multiple potential points of failure in a sequence of data loading pipelines. At each step, the loading pipeline pulls data in from the source (INCOMING DATA 1, 2, 3, …, *n*). The corresponding data in the database, represented by boxes with striped backgrounds, are removed in preparation for the newly imported data to be loaded (boxes with solid backgrounds). A pipeline failure, denoted by a red X and the absence of the corresponding box within the database icon, prevents the sequence from continuing until the problem has been fixed and that pipeline has run successfully. If there are additional issues at any subsequent steps—either pre-existing or potentially introduced by a previous repair—the load fails again until those are also fixed. In this way, drop-and-reload introduces the risk that substantial sets of data could be dropped and not reloaded, compromising data integrity, and that data releases to the public might need to be delayed.

The major disadvantage of drop-and-reload is that efficient loading of large datasets can pose problems, especially when the incoming data change. For example, there is a risk that large subsets of data could be dropped and not replaced if a pipeline fails midstream, necessitating either a database rollback or an emergency fix and reinitialization of the pipeline. Also, as new data sources are added, pipeline dependencies can change, and pipeline workflows can quickly become complex and tedious to maintain. Because of these dependencies, data releases tend to be delayed when problems with incoming data are detected. Figure 1 shows how such dependencies in a relatively simple sequence of drop-and-reload pipelines introduce multiple points of failure, all of which must be addressed before the entire data loading process can complete.

The first process to import rat gene records from the National Center for Biotechnology Information (NCBI) Gene database into RGD was largely a drop-and-reload pipeline run once or twice a year at most. Although RGD IDs were maintained, all of the resident data corresponding to that being imported were stripped from the database to allow data to be reloaded from the source. The process involved a preliminary load into a temporary database, BLASTing all incoming sequences against those in the RGD datastore, extensive manual checking by curators, and a second load into RGD proper. Being both time- and resource-intensive, it took an average of 2 weeks to complete, and the infrequency of updates meant that RGD was largely out of sync with the data at NCBI for the same genes.

RGD’s first automated pipelines, written in 2006, substituted ID matching, specifically of the RGD ID, NCBI Gene ID, and at least one sequence ID, for sequence alignments (Supplemental Figure S1, Rat NCBI Gene Bulk Load Flowchart). The load process required 2 days rather than 2 weeks and therefore could be run on a weekly basis so that RGD would remain in sync with NCBI. If an incoming record matched an RGD gene on only one or two of the three required criteria, the gene was exported into a “conflicts” log file, which was then reviewed by a single curator who made corrections as necessary so that the genes in question could be updated in a successive load. If none of the three matching criteria were found in RGD, the gene was considered “new” and was assigned a new RGD ID and loaded into the database as a new record.

These first pipelines were largely drop-and-reload, but the need to maintain gene RGD IDs as persistent identifiers required more of a hybrid approach. The basic gene records, their associated RGD IDs, and their associations with manually curated annotations remained in place. However, once a match was made between an incoming record and one existing at RGD, all the associated attributes that had originally come from NCBI, such as position information and GenBank and RefSeq identifiers, were removed from the RGD gene record and reloaded from the incoming data. On the other hand, pipelines which handled other types of imported data such as protein-associated external database IDs from UniProtKB were fully drop-and-reload. This was considered likely to be the fastest option and the best choice for keeping the data fully in sync with the originating resources. However, later work demonstrated that the biggest bottleneck in the process was not the matching of the records but rather the queries to the database and the process of loading data into it. This prompted a change in strategy from drop-and-reload to incremental updates.

## The incremental update paradigm

**Figure 2.**
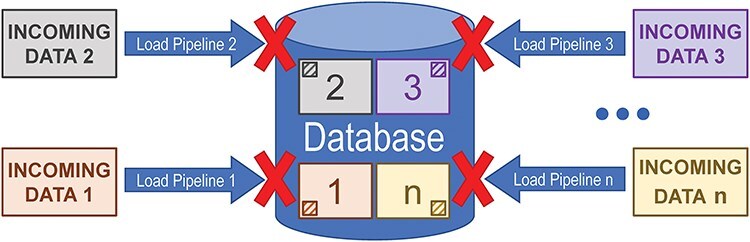
Diagram illustrating how only updating a subset of the data in a database when that subset changes at the source can prevent a failure in one pipeline from interfering with other data loading pipelines that run against the same datastore.

Incremental updates utilize an “always-present” persistent datastore. Data are loaded via any number of pipelines which are generally independent of each other. Usually, only a small part of the data will be updated during any given pipeline run. As shown in Fig. 2, because the pipelines operate relatively independently, a failure in one pipeline will not prevent the other pipelines from running and will generally not interfere with data releases.

With these considerations in mind, RGD developers rewrote the early drop-and-reload and hybrid pipelines and have developed all subsequent pipelines using an incremental update approach. This fundamental change to incremental updates reduced the time required for a full load of genes for a species from 2 days to 2 h. Additional improvements have reduced that even further. Although extensive updates are occasionally required, e.g. when there is a new genome assembly for a species such that nearly all the genes are assigned new positions, most of the data do not change substantially from week to week. Therefore, it has proven to be much faster to quality control (QC) the entire incoming dataset but then only use the small proportion of it that is new or changed to update existing records in the database rather than to load the entire dataset into the database.

To illustrate using NCBI Gene as an example, data for genes are imported weekly from the NCBI database. These include, but are not necessarily limited to, position data, external database links, and transcript/gene model definitions (i.e. exon, coding sequences, and untranslated regions positions for each transcript of the gene). If one external database ID was changed in the incoming record, in the scenario where gene records and their associated data were dropped and reloaded *de novo* each time, a minimum of five tables in the database would need to be accessed to reinsert the values, most of which would not have changed. On the other hand, in the incremental update scenario, only a single update to a single table is required.

RGD maintains three independent environments—development, staging (referred to as “pipelines” or “curation”) and production databases and websites. Data releases from the RGD pipelines/curation database to the public-facing/production database generally occur automatically on a weekly basis. Between releases, each data loading pipeline runs one or more times a week, updating the data within its area of responsibility. To prevent pipelines from creating or propagating errors, all pipelines employ a “short-circuit” paradigm. For instance, if the format or structure of the incoming data has changed so that the pipeline cannot read and interpret the data, or if at any point in the process, the pipeline is unable to access the database, the pipeline fails and no more processing is done. Though infrequent, when such failures do occur, the problem can be prioritized and repaired based on the urgency of the problem and developer availability. In those cases, although a small subset of the data might briefly be out of sync with the originating database, the pipelines run essentially independently, so there is little or no impact on the functioning of other pipelines.

Although RGD’s implementation of the incremental update paradigm has proven to be very successful, there are some disadvantages. Because for most RGD pipelines, comparisons between the data from the database and the incoming data are done in memory, i.e. the data are queried from the database in bulk and stored in memory to be used for comparisons, for larger datasets the memory requirements can be substantial. For example, RGD imports variant records from the European Variation Archive (EVA) for rat, mouse, dog, pig, and green monkey (Table 1). Each EVA load brings in almost 260 million records. Although the local RGD pipeline server has 256 GB of RAM configured, this is not enough to hold both these incoming variants and the variant records currently in RGD in memory to allow for an incremental update of the entire dataset. To accommodate the memory limitation, the EVA load pipeline splits the data on the species, assembly, and chromosome and runs separately for each segment of the data. Although larger and/or more server instances could be set up in a cloud environment, at present, the expense has not been considered justifiable since the local hardware is available and a relatively easy work-around for the memory limitation has been implemented. As datasets get larger, this decision will need to be revisited on a regular basis.

Another challenge is to determine what data currently in the local datastore have been removed from the incoming dataset. Safeguards are required to prevent the accumulation of “stale” data. For example, when a set of ontology annotations are imported, the pipeline attempts to match each incoming annotation with one already existing in RGD. If a match is found, the last modified date on the RGD annotation is set to the time the pipeline “touched” that annotation. This incrementation occurs regardless of whether differences in the incoming data require that updates be made to the RGD record or the pipeline determines that the annotation has not changed and no updates are required. At the end of the pipeline run, all the annotations attributed to that pipeline, i.e. that had been created by the pipeline at any point in the past, are checked. Any annotations where the last modified date is before the start of the most recent pipeline run are designated as having been removed from the source database and are deleted from RGD.

Although this functionality ensures that stale annotations are removed, it introduces its own challenge. If the imported data were incomplete, for instance, because of a problem at the source or because the data transfer was not complete during the import, a large proportion of the annotations from that source could be deleted. To prevent this, a limit was imposed on the percentage of data from a given pipeline that could be automatically deleted. When the percentage to be removed exceeds 5% of the total data derived from that pipeline, the step to delete the purportedly obsolete data fails. No data are deleted so that the developer and other stakeholders can determine the proper course of action to correct the problem.

## Standardization of pipeline infrastructure facilitates the development of new pipelines as well as maintenance and improvement of existing pipelines

RGD utilizes a relatively small team of approximately three full-time effort developers to support all of RGD’s development needs, including database and website development and maintenance, development of new software tools, and data handling (import, export, and QC). The use of a uniform coding platform—specifically the use of the Java coding language (https://www.java.com/) [53] and the Spring framework (https://spring.io/) [54]—maximizes the effectiveness of team members, allows the team to focus on programming, and ensures that, when necessary, maintenance and repair of code can be performed by any member of the team, not just the original developer. Code transparency and ease of deployment are enforced through the use of the standard source code repository GitHub (https://github.com/) [55] and the Jenkins build server (https://www.jenkins.io/) [56].

Consistency and efficiency have been both promoted and reinforced by the ongoing development of a core shared Java software library (https://github.com/rat-genome-database/rgd-core-library) used by all applications across the RGD ecosystem. This library, which is updated and expanded on an ongoing basis, contains a software domain layer to represent biological objects and data access objects (DAOs) that communicate with persistent storage, common biological logic, and validation logic. Biological object creation, retrieval, updating, and deletion are all managed by the shared library. This simple shared architecture has provided many advantages to the project. All developers are trained on, add to, and actively use these libraries so that expertise is seamlessly shared throughout the team. This improves cross-training and allows any developer to take part in maintenance. Application development speed and efficiency are increased due to the lack of duplication across the ecosystem. It is common for a new pipeline or application to require logic that has already been developed. These shared libraries encourage code reuse and make that logic easily accessible, increasing consistency in development. Since all RGD applications have access to and use the same library, the same result is rendered independent of the application making the call. When code is added to the library, that new functionality is available to all applications and all developers. If a new biological object or method is needed that is not currently represented, any developer can add to the library, ensuring that the library evolves over time. The use of a shared domain and DAO layer improves the readability of all applications using the library and removes persistence logic from the application. This also decouples software applications from the persistent store, allowing the physical storage to be changed without affecting downstream applications.

In a similar way, a consistent architecture has been created to standardize pipeline structure. Focusing on relatively few well-established technologies reduces complexity and creates a stable environment. Reuse of code means that developers focus on the higher logic that determines the functionality of a specific pipeline, not the basic elements that are shared across pipelines. In addition to the use of Java and Spring, the Bash scripts used for running the pipelines and standardized logging scripts are replicated across the pipelines rather than these basic functions being written each time. The use of the Gradle Build Tool (https://gradle.org) [57] for building automation simplifies the management of dependencies for the pipelines.

To streamline pipeline maintenance, wherever possible, the properties that are essential for pipeline functionality but that can be variable between pipelines and/or are expected to be updated periodically are encoded in property files rather than being hard-coded in the pipeline itself. Such properties include, for instance, the RGD species for which a pipeline is importing data (“speciesProcessed”) and locations of source data files. Ensuring that these variables are specified only once for a pipeline and in a location that is consistent across pipelines makes them easy to find and change.

**Figure 3.**
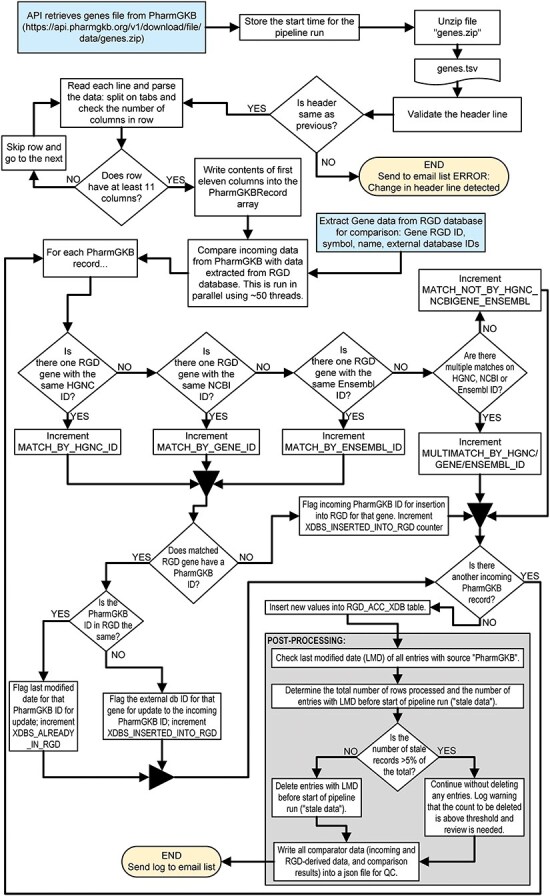
Flowchart illustrating the steps of the PharmGKB pipeline which associates PharmGKB identifiers with human gene records in the RGD database.

**Figure 4.**
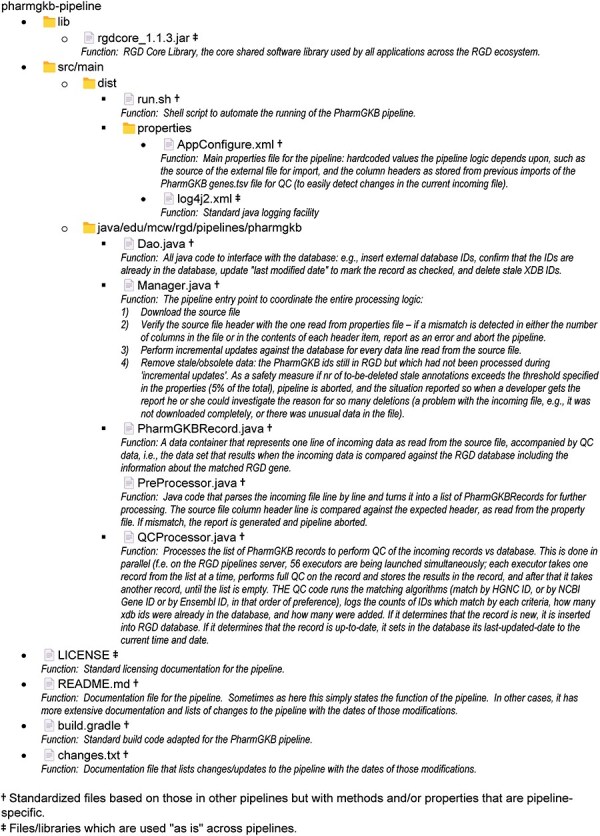
The list of folders and files that constitute the PharmGKB pipeline with short descriptions of the function of each file in the list, presented as an image.

A simple example of an RGD pipeline is the one which imports external database links for the PharmGKB database [58, 59] and associates them with RGD genes. The code is publicly available and can be found at https://github.com/rat-genome-database/pharmgkb-pipeline. As detailed in Fig. 3, this relatively simple pipeline imports a tab-delimited file containing all of the PharmGKB-ID-to-gene associations from the PharmGKB website. The program parses the file and splits the lines into an array. Where possible, each gene in the file is paired with an RGD gene by matching the incoming HGNC ID, NCBI Gene ID, or Ensembl Gene ID (in that order of preference) to a corresponding external database ID for a gene in RGD. The pipeline then either confirms that the PharmGKB-ID-to-RGD-gene relationship already exists or adds that to the RGD database. This pipeline contains all the architectural elements that are shared among RGD pipelines and illustrates RGD’s standardized architecture, code reuse, and QC practices such as setting an upper limit to the number of stale IDs (i.e. IDs which are no longer found in the incoming file) that can be deleted. Figure 4 shows the list of files and folders contained within the PharmGKB Pipeline GitHub repository, with information about the function of each file in the list. Files marked with a double dagger (**‡**) icon are files which are used “as is” across pipelines. Those marked with a dagger (**†**) icon are standardized files derived from or based on those in other pipelines but with methods and/or properties that are pipeline-specific.

Pipeline development is carried out initially on the development code branch where imports can be checked and the integrity of the data can be confirmed. The version control tool Git is used for source control. Initial code development is done on the developer’s local system. When ready for testing, it is submitted to the dev source control. The code is compiled, checked against the RGD core library to ensure that it matches the applicable domain objects and database schema, and then automatically deployed to the development/staging server. If any of these checks are out of sync, the deployment fails. Once successfully deployed, the pipeline jobs are configured to run daily. Daily runs are repeated for several days, or even weeks, prior to the code being moved to production. Data integrity tests are run each time the pipeline executes, and notifications are sent on failure. In addition to automated integrity testing to compare incoming data with data in the database after loading, the pipeline developer reviews the logs and summaries and runs additional manual queries both before and after the pipeline runs to ensure that the data were handled correctly. If anomalies are found, the developer corrects the code and tests it again. When needed, the development team will participate in code reviews for cross-training, to ensure code reuse where possible and beneficial and encourage collaborative problem-solving. Deployment of code from the development system to production must be manually triggered and only takes place after a history of successful and accurate runs. Because the RGD production environment (database structure, etc.) is essentially identical to the development environment, no code changes are needed between development and production. Code can be moved seamlessly from development to the internal-facing pipelines servers and then in turn to the public-facing production servers.

## Harmonization of data

Table 2 provides a list of the automated pipelines which are currently in use at RGD, almost half of which are data import pipelines (see Table 1 for information about the resources from which RGD’s import pipelines retrieve data). Because the data types handled by these vary and even data of the same type can come from multiple sources, each of which may have its own formats and practices, the incoming data are rarely standardized or harmonized. Very often, there is a requirement for initial mappings, e.g. between ontologies, and/or for the determination of a set of match criteria that can be used to ascertain if an incoming record has already been loaded into RGD. In addition, because of differences in the incoming data, the process of data harmonization, and in many cases standardization, must be done as part of the loading process. With respect to the fields that can be used to determine the uniqueness of a record and to map incoming records to existing records, the determination can generally be done at the requirements-gathering stage and rarely needs additional hands-on review.

**Table 2.** List of RGD automated pipelines

Pipeline name	Pipeline short description	Pipeline type
**RGD import pipelines**		
array-id-import-pipeline	Import array IDs with synergizer	Import
biocyc-pipeline	Import BioCyc pathway IDs and assign them to RGD genes	Import
cellosaurus-pipeline	Import cell lines from Cellosaurus at Expasy	Import
chromosome-pipeline	Load chromosome and cytoband information	Import
clinvar-pipeline	Load variants from ClinVar into RGD database; create disease and human phenotype (HPO) annotations for variants and their associated genes	Import
cosmic-pipeline	Generate external database IDs to COSMIC database (https://cancer.sanger.ac.uk/cosmic) for active human genes	Import
ctd-chem-drug-pipeline	Import gene–chemical interaction annotations from CTD database	Import
ctd-disease-annotation-pipeline	Load disease annotations to genes based on CTD FTP files	Import
db-snp-pipeline	Load human variants from DB_SNP into db	Import
ensembl-data-pipeline	Load genes and transcripts for all RGD species, plus protein IDs, from Ensembl into RGD	Import
epd-pipeline	Load rat, human, mouse, and dog promoters from EPD	Import
eva-pipeline	Load variants from EVA for rat, mouse, dog, pig, and green monkey	Import
expression-load-pipeline	Load experiment values from Expression Atlas	Import
*gene-description-pipeline*	*Generate gene descriptions for rat, mouse, and human based on automated gene synopses imported from the Alliance of Genome Resources merged with automated gene synopses from RGD for annotation types not covered by the Alliance descriptions*	*Import*
gene-loading-pipeline	Load gene models from NCBI	Import
go-nonrat-annotation-pipeline	Load/update GO annotations for all species from EBI’s GOA database and the GO Consortium	Import
go-rat-annotation-pipeline	Import GO annotation for rat from EBI	Import
gwas-catalog-pipeline	Import the data from the GWAS Catalog	Import
hrdp-variant-load-pipeline	Load the HRDP Variants into Variant Visualizer	Import
human-proteome-map-pipeline	Load Human Proteome Map IDs for human genes	Import
imex-interactions-pipeline	Load protein interactions from IMEX database	Import
mirna-pipeline	Load miRNA data, previously from miRGate and miRBase, now only from miRBase	Import
mouse-disease-annotation-pipeline	Import disease annotations from MGI	Import
mouse-qtl-pipeline	Load QTLs for mouse using files from MGI FTP site	Import
ncbi-gene-pipeline	Load nucleotide and protein sequences for genes, all species, from NCBI	Import
omia-pipeline	Create annotations from OMIA	Import
omim-annotation-pipeline	Load OMIM IDs and then create OMIM disease annotations	Import
ontology-load-pipeline	Load all ontologies used by RGD	Import
*ortholog-pipeline*	*Load ortholog assignments from HGNC, HCOP, NCBI, and the Alliance of Genome Resources*	*Import*
pharmgkb-pipeline	Import PharmGKB external IDs from files downloaded from PharmGKB website	Import
phenogen-pipeline	Load external database IDs to PhenoGen database at UC Denver	Import
phenominer-load-pipeline	Load data into Phenominer tool	Import
phenotype-annotation-pipeline	Phenotype annotation pipeline, for mouse and human genes	Import
ratgtex-pipeline	Load external RatGTEx external IDs into RGD	Import
rat-mutation-sequences-pipeline	Load rat mutation sequences to database to generate variant pages informatically from large-scale submissions of mutant strain/allele/variant data (Run on demand)	Import
rna-central-pipeline	Import RNACentral IDs as “external database IDs” for all noncoding genes in RGD to generate links to RNACentral database	Import
rna-seq-pipeline	Import records from GEO and do preliminary text mining, extraction, and natural language processing to suggest values for RNA-Seq metadata	Import
small-molecule-pathway-pipeline	Create pathway annotations based on data from Small Molecule Pathway Database	Import
strain-rrrc-pipeline	Bulk loading of strains from the Rat Resource & Research Center (https://www.rrrc.us/)	Import
synteny-load-pipeline	Create synteny blocks for all species	Import
uniprot-pipeline	Import external db IDs, protein objects, and sequences from UniProtKB	Import
variant-load-pipeline	Load rat strain data from VCF files into RGD database and precomputes data for use by Variant Visualizer tool	Import
**RGD export pipelines**		
*alliance-submission-pipeline*	*Generate and submit files for rat and human to the Alliance (* https://www.alliancegenome.org/)	*Export*
europe-pmc-pipeline	Generate an XML file of article-to-data-object associations to send to Europe PMC	Export
ftp-file-extracts-pipeline	Generate weekly files for RGD FTP/download site	Export
goc-annotation-pipeline	Generate GO annotation files in GAF format	Export
**RGD data indexing pipelines**		
elastic-search-indexer-pipeline	Create elastic search indexes	Data indexing
full-annot-index-pipeline	Update FULL_ANNOT_INDEX table: big auxiliary table used to speed up many annotation queries used by RGD tools	Data indexing
full-record-index-pipeline	Precompute summaries from experiment record data into FULL_RECORD_INDEX table for subsequent use by Phenominer tool	Data indexing
gene-loci-pipeline	Populate GENE_LOCI table used by Variant Visualizer tool	Data indexing
interaction-counts-pipeline	Compute “interactions count” for genes and proteins and store it in INTERACTION_COUNTS table	Data indexing
ontosolr-pipeline	Index abstracts and tags for OntoMate’s solr index	Data indexing
phenominer-annotation-index-pipeline	Populate table PHENOMINER_RECORD_IDS	Data indexing
statistics-archiver-pipeline	Generate comprehensive statistics for objects in RGD database and store it; run weekly	Data indexing
variant_indexer_rgd	Index variant data for search	Data indexing
search-indexer-pipeline	Build general search index, in the Oracle database. Mostly legacy but some of the resulting index data are still used by existing processes	Data indexing
**Internal RGD pipelines that process data (e.g. propagate ontology annotations across species or data types)**		
gff3-pipeline	Create gff3 files to be loaded into RGD JBrowse instance	Data processing
gtex-pipeline	Create GTEx external database IDs for human genes, based on Ensembl Gene IDs, and if these are not available, based on gene symbols	Data processing
gwas-annotation-pipeline	Create QTL and variant annotations from imported human GWAS data	Data processing
jbrowse2_pipeline	Build and deploy JBrowse2	Data processing
mutant-strain-annotation-pipeline	Propagate disease and phenotype annotations from strains to alleles and genes	Data processing
object-mapper-pipeline	Calculate/update assembly map positions for strains, QTLs, and markers	Data processing
orthology-paf-pipeline	Generate PAF files for Jbrowse2 based on orthology	Data processing
phenominer-expected-ranges-pipeline	Calculate strain expected ranges for clinical measurements	Data processing
qtl-rso-annotation-pipeline	Assign RS ontology IDs to QTLs based on rat strains used to calculate the QTL	Data processing
strain-vt-annotation-pipeline	Propagate VT annotations from QTLs to associated strains	Data processing
transitive-annotation-pipeline	Create ISO annotations for all public species in RGD based on manual gene annotations	Data processing
transitive-ortholog-pipeline	Generate transitive “orthologs” (i.e. homolog relationships) for all species based on HUMAN orthologs	Data processing
vcmap-loading-pipeline	Format and load data for RGD’s VCMap tool from UCSC’s synteny data files	Data processing
vep-pipeline	Annotate variants in RGD through the VEP tool from Ensembl	Data processing
**RGD internal QC pipelines**		
data-qc-pipeline	Generate general purpose QC reports on data integrity	Internal QC
ensembl-pipeline (QC)	Download genes from Ensembl and compare them against genes in RGD. Produce report of differences	Internal QC
fix-duplicate-ref-rgd_ids-pipeline	Fix the problem of duplicate PubMed IDs for references	Internal QC
hgnc-pipeline	Handle obsolete HGNC IDs for human genes	Internal QC
nomenclature-pipeline	Populate entries in the nomenclature review tool—check gene symbols/names for rat against the human and mouse orthologs, where different add them to the tool for review	Internal QC
perpetuate-nomenclature-to-strain-pipeline	Report strains with nomenclature that do not match the corresponding gene or allele nomenclature for review by a curator	Internal QC
phenominer-qc-pipeline	QC scripts to report problems with the integrity of the data used by Phenominer tool	Internal QC
protein-qc-pipeline	Check for problems with amino acid sequences calculated from reference-assembly derived trascript sequences for Variant Visualizer tool	Internal QC
qtl-qc-pipeline	Check for QTL records with multiple associated Clinical Measurement terms and/or multiple Vertebrate Trait terms	Internal QC
reference-update-pipeline	Reference update pipeline: (i) fix duplicate RGD IDs for each reference and (ii) import missing references for PubMed IDs created within last few days	Internal QC
strain-rso-annotation-pipeline	QC utilities for Phenominer data: Strain ontology	Internal QC
strain-synonym-fix-pipeline	Replace “||” and “,” separators with “;” in ALIASES table for aliases of type “old_strain_symbol” and “old_strain_name”	Internal QC
update-objects-in-full-annot-pipeline	Update names and symbols of genes/strains/qtls/variants in FULL_ANNOT, to keep them in sync with tables GENES, STRAINS, QTLS, VARIANTS	Internal QC
update-secondary-go-id-pipeline	For GO annotations, replace secondary GO IDs with primary GO IDs	Internal QC
update-terms-in-full-annot-pipeline	Internal QC pipeline for FULL_ANNOT table: update term names and aspects	Internal QC
**RGD notification pipelines and legacy pipelines**		
notification-pipeline	Send notification e-mail to outside users who subscribe to data object or ontology term updates (“watchers”)	Notification
strain-files-pipeline	Send an e-mail with new strain files from the past week	Notification
rgd-pipelines-lib	Java library: a framework to process streams of data in multiple parallel threads. Not used since Java Streams was implemented	Legacy
portal-processing-pipeline	Precompute data needed by disease portals. No longer needed since disease portal code was completely rewritten. Portals now use the structure of the ontologies to query the associated data on the fly without this type of preprocessing	Legacy

List of RGD pipelines with the name of each pipeline, a short description of its function, and the pipeline type, i.e. whether the pipeline imports data, exports data, creates indices, processes data, performs QC functions, such as finding inconsistencies and either producing a report or correcting the errors, sends internal or external notifications, or is a legacy pipeline that is no longer used. Pipelines which are either partially or fully dedicated to importing data from or exporting data to the Alliance are highlighted with text in italics.

In certain cases, however, the process of review and harmonization needs to be ongoing. For example, RGD imports human clinical variants and their associations with medical phenotypes from the ClinVar database [30]. ClinVar uses a number of vocabularies to represent the phenotypes associated with a given variant, whereas RGD uses the human Disease Ontology (DO) [12, 60, 61] for disease annotations. When RGD first began to import ClinVar data, a major curation project was required. Both automatic matching of ClinVar conditions to DO terms by text and/or IDs and manual review of those matches were performed, and, where needed, additional manual assignment of correspondences was undertaken. Now, as variants are imported, the original text of the associated medical conditions for each variant is stored in a free text field in the variant record to maintain consistency with the source data. Incoming textual phrases that had been matched to terms in the RGD disease vocabulary are used to make DO annotations to the RGD variant records and associated genes. “Conditions” that have not been matched to DO are included in the variant record and are documented by the pipeline. Since the DO is continually being updated, these unmatched condition terms are reviewed periodically to determine if appropriate matches can be found so that DO annotations can be made. This harmonization is required to allow annotations from the ClinVar data to be seamlessly incorporated and utilized with disease annotations from RGD and other groups.

As a founding member of the Alliance, RGD curators and developers have been involved in the challenging process of data harmonization across the MODs [8]. During the development of Alliance data models, each working group reviews the data related to their area of expertise found at the MODs, beginning with the data and metadata elements that are shared across most or all of the MODs. Once the basic model is in place, it can be expanded to include elements that are specific to one or a few research communities. Data quartermasters then use the harmonized model to structure their own data for submission. Because the standardization of the data to conform to the Alliance model is carried out at the MOD before submission, the process may seem to be different from RGD’s internal process of harmonization of incoming data, but the steps to determine what is shared and what, if anything, needs to be adjusted with respect to the incoming data, the data model, or both are essentially the same.

In addition to participating in the development of the Alliance data models, as one of the Alliance Knowledge Centers, RGD both exports data for integration into Alliance Central and imports data from Alliance Central. The pipelines which interface with Alliance Central are noted with italicized text in Table 2. The single set of export pipelines (see the first entry in the RGD export pipelines section of Table 2) produces standardized JSON-formatted files according to the corresponding LinkML models for the following data:

- Rat and human gene records
- Rat strain/affected genomic model records
- Rat phenotypic allele records
- Rat variant records for phenotypic alleles
- Manual disease annotations for rat and human
- Imported disease annotation from Online Mendelian Inheritance in Man (OMIM) for human (“OMIM via RGD” annotations)
- Manual phenotype annotations for rat and human
- Imported human phenotype annotations from the Human Phenotype Ontology (HPO) database
- Reference records for all publications used for curation
- Additional files in other standard formats are also exported by the pipeline
- Rat variant records (variants from whole-genome sequencing of rat strains, submitted as VCF files)
- Human ClinVar variant records (submitted as VCF files)
- Multiple rat and human GFF3 and VCF files for Alliance JBrowse 2 tracks
- Human Gene Ontology annotations (GOAs) assigned to genes based on UniProtKB annotations assigned to proteins.

All these files are made publicly available on RGD’s download site at https://download.rgd.mcw.edu/data_release/agr/.

RGD’s data quartermaster and developers work closely with other members of the Alliance to ensure that RGD’s data are submitted correctly and successfully. The Alliance tracking system for data loads makes it straightforward to see what files have been submitted, whether they loaded successfully, and, when not successful, what caused the failure.

## Curator involvement

RGD curators work closely with developers throughout the process of pipeline development and maintenance. When the decision is made to import a new dataset, at least one curator reviews the data at the source datastore to determine the requirements for integrating the data into RGD’s schema and architecture. Although RGD curators participate in all types of data curation (disease, phenotype, Gene Ontology, etc.), each has a specific area of expertise. Selection of the curator(s) to participate in the integration of a specific datatype is determined based on this. In addition to the specification of requirements, the curator will collaborate with developers to establish a data model and determine if changes to the database schema are required. During the pipeline development, curators manually check the incoming data against the records at the source database and review the log files and any error reports to help developers troubleshoot the data processing. Once an initial version of a pipeline has been finalized, both the consulting curator and the pipeline developer continue to review the logs and work together to solve issues when they arise.

## Improvements in speed and efficiency

As the size of data imports and the number of pipelines being run increased, pipeline speed and efficiency have become paramount. While the first pipeline for loading gene records from NCBI was single-threaded, changes to multithreading and in-memory processing using a custom library for processing multiple streams of data reduced the time to update the metadata for ∼25 000 rat gene records from 2 h to ∼30 min (∼14 genes/s). Implementation of Java Streams further increased both the speed and efficiency of data loads and updates significantly. To illustrate this, new reference genome assemblies were recently released for both bonobo (NHGRI_mPanPan1-v2) and rat (GRCr8). When the annotations for a new assembly are first imported, essentially all the genes for the corresponding species receive new position data, and hundreds or even thousands of new gene records are added. In these two cases, RGD’s NCBI Gene import pipeline processed ∼42 000 gene records for bonobo in ∼20 min and 121 000 gene records for rat in ∼1 h, giving a consistent throughput of ∼35 genes/s across both gene sets. Recent testing on the PharmGKB pipeline described previously with and without caching and multithreading demonstrated that for this smaller pipeline, there was no significant difference in run time based on whether the data were queried from the RGD database and cached locally for the comparisons or not. Not surprisingly, however, the difference between running the pipeline multithreaded compared to running it single-threaded increased the speed more than five-fold (Table 3).

**Table 3.** Results of PharmGKB pipeline speed test After downloading the source genes.zip file, the RGD PharmGKB pipeline was run with and without locally caching the data for genes and associated external database IDs from a single bulk query for use in the gene matching steps of the pipeline (caching), and with and without leveraging Java Streams across multiple cores (multithreading). Results show a minimal decrease in the time elapsed for the full pipeline run when caching was included. Multithreading, with or without caching, reduced the run time approximately five-fold

PharmGKB pipeline elapsed time (seconds)		Multithreading	
		False	True
**caching**	**False**	63	12
	**True**	62	11

Note that these times are only for processing already downloaded gene records. Because of the limitations on the amount of data that can be queried from the NCBI database at one time using the E-utilities API, it takes longer to download the records than it does to process them. To minimize the time required to download, for instance, a full set of genes for a species, RGD has developed an adaptive download process. When the download begins, the pipeline queries for the list of IDs for genes that have been updated in the interval since the last time the pipeline ran (usually a week). The pipeline will then start by trying to download the records for a relatively small number of those genes. If that request finishes successfully, the next request will be for proportionally more records. Each time the query is successful, the pipeline will request a larger number of records until the request fails. When the request fails, the pipeline will determine which genes from the last request were not returned and reincorporate those gene IDs into the remaining list of genes to be downloaded. The list is shuffled, and the pipeline reduces the number of gene IDs in its next request by half. If that request is successful, the process begins again, increasing the number of gene IDs in each request until a failure. The cycle is repeated until the records for all the genes requiring updates have been downloaded. This strategy minimizes the amount of time required for the download while respecting the necessary limits imposed by source databases like NCBI.

## Pipeline logging

Earlier RGD pipelines stored extensive and detailed information about each piece of imported data in dedicated database log tables and made it available to curators for manual review via a specialized tool. Although helpful for the early pipelines while the system was being developed and at a time when the data needed much more checking and many more corrections, as the data and the pipeline system have matured and stabilized, the level of detailed oversight required has substantially reduced.

RGD currently uses a simplified system to log pipeline activities and notify stakeholders. For all the major pipelines, changes made by the pipeline are entered into monthly log files stored on the pipelines server. These files are not available publicly but can be used internally by the development team when needed. Both individual changes and summary data are logged, as well as any exceptions that happened during the pipeline run. On occasion, an anomaly is found in data loaded by a pipeline. To ensure that the data in RGD can be checked against the exact input data file, all the incoming files are stored for a period of a year. Most of the older files are deleted periodically, but at least one file that represents a full load of the applicable data from each year is maintained. These previous copies of incoming data are useful for troubleshooting problems with downloaded data, such as a significantly lower or higher amount of data, and changes in the incoming file format.

After each pipeline run, a summary e-mail is sent to all stakeholders for that pipeline, i.e. the pipeline developer(s), any curators who were involved in the development of the pipeline, and members of the RGD management team. After a successful run, the summary e-mail contains information about the number of incoming records; how many of those could be matched to data in RGD; how many objects or attributes were added, removed, or updated; how many objects or attributes were checked but required no updates; and the pipeline run time. Much of the information in the summary e-mails is shared across all the pipelines, but each also has its own specific data included to help with troubleshooting that particular pipeline. If a pipeline fails, the notification e-mail contains summary information if any exists with a statement that the pipeline failed, the reason for the failure, and a copy of any error/exception messages that occurred. With this system, one person can easily review all the summary e-mails each week. Any issues that arise can be quickly and efficiently triaged. Although data problems might require the input of a curator/biologist, purely technical issues can be resolved by the pipeline developer.

One example of when the simplified logging helps stakeholders recognize an unusual event in a pipeline run is during the addition of position data on a new assembly. RGD is not the source of truth for genome annotations for rat or any other species in the database, but rather relies on gene position data and gene model definitions which are imported by the NCBI and Ensembl Gene pipelines. Using rat as an example, when data become available for a new rat assembly, the NCBI Gene pipeline detects an unrecognized genome assembly. The stakeholders are notified by the logging e-mail that a new assembly has been detected. The pipeline developer confirms that the file contains data for a new rat assembly and that there are an unusually large number of updated records in the incoming dataset. A new assembly object is added to RGD, and the pipeline is rerun to load new position data onto RGD’s gene records as well as add new gene predictions that are specific to the incoming assembly. E-mail notifications of changes in the incoming data and unusually large numbers of incoming records make it immediately clear to the stakeholders that a substantial change was made at the originating database. The data can be quickly checked to establish what is different and take steps to either handle the new data or correct a problem if one exists. Once the data are loaded, RGD curators collaborate with those at RefSeq to clean up “stale” gene models that were predicted for the previous assembly but do not map to the new one. In the case of gene records and any other data object to which an RGD ID has been assigned, the objects are either withdrawn or retired rather than being deleted outright. For rat, this process of manually checking and removing records when needed can extend for several months after a new assembly is released. However, the automated pipelines and the simplified logging system make the initial addition of data for a new assembly relatively seamless.

## Pipeline documentation

Code documentation is generally considered an essential part of software development [62]. It is, for instance, listed among the best practices for code quality assurance by the UK Office of National Statistics (https://best-practice-and-impact.github.io/qa-of-code-guidance/intro.html). Rather than maintaining a separate documentation stack, RGD has made the use of self-documenting code a standard practice. Variables and methods are given human-readable names that clearly denote their function. Comments are added to provide information about what the code is doing, why it is written the way it is, and/or how to use the code. README files are also used to document the basic requirements for each pipeline.

## Discussion

A recent press release from the National Institutes of Health quoted Dr Erin Ramos, deputy director of the NHGRI Division of Genomic Medicine, as saying “Multi-omics studies are at the forefront of biomedical research and promise to advance our understanding of disease onset and progression” (https://www.genome.gov/news/news-release/nih-awards-dollar-50-3-million-for-multi-omics-research-on-human-health-and-disease). Likewise, in recent years, a number of research and review articles have provided strong arguments in support of the use of diverse rodent and nonrodent models for the study of human disease (e.g. [4, 63–71]).

RGD at its inception was created as a resource for genetic and phenotypic data for the laboratory rat, with information about mouse and human for comparative studies. In the 25 years since then, the focus has expanded to include data across the omics spectrum and across 10 mammalian species. This expansion has been facilitated through the development of standardized pipelines for data import, export, and QC. Standardization of the pipeline infrastructure speeds up development of new pipelines, simplifies maintenance of existing pipelines, and supports diversification of both data types and data sources. The development process is focused on the higher logic required to integrate generally nonstandardized data rather than developers needing to spend unnecessary time in creating the fundamental pipeline infrastructure from scratch each time.

As pipelines have been developed, maintained, and improved, the following practices have proven efficacious in simplifying and accelerating the process:

- For large data loads and more complex data structures, an incremental update paradigm proved to be faster, more reliable, more sustainable, and more extensible than a drop-and-reload paradigm.
- The slowest part of the data loading process was found to be database operations such as querying, reading from, and writing to data tables. Therefore, performance was significantly increased when data were extracted in bulk into memory for comparisons versus querying for and individually updating each piece of data.
- Putting all changeable, hard-coded parameters into properties or configuration files gave developers a single, standardized location for these arguments, substantially simplifying maintenance and updates.
- Creating a standardized code template facilitated the process of writing, maintaining, and updating data loading pipelines. Once a new developer has become familiar with the template, they are able to both maintain any existing pipeline and write new pipelines with little or no additional training.
- The problem of “stale” or “leftover” data (i.e. items which are no longer present in the source data but still exist in the local datastore) was solved by assigning each item a “last modified” date and incrementing that date whenever the data item is compared to an incoming record to determine whether the data have changed. At the end of any run intended to update every record from a given data source, all records where the last modified date remains unchanged are no longer in the source data and should be removed from the local datastore.
- Incorporating processes to ensure data integrity such as employing a short-circuit paradigm that aborts the pipeline when fundamental changes are detected in the source data and setting limits on the number of records that can be removed from the database as being “stale” at any given time has prevented issues that could have required a database rollback or other rapid and immediate work to correct.

MODs, in general, and RGD, specifically, are committed to the integration of diverse data types to support translational, multiomics studies of human physiology and disease. To minimize duplication of efforts while providing a more comprehensive dataset, data are moved into and out of the local datastore using automated pipelines. Import pipelines incorporate, standardize, and harmonize data from multiple sources while maintaining the data provenance, so users can always go back to the source for more information. Export pipelines enable large-scale sharing of these data with researchers and with other databases in keeping with the FAIR and open data principles. QC pipelines ensure consistency within and across datasets. Improvements to and standardization of the pipeline code make the pipelines more robust and ensure the efficiency of development and maintenance of these important components of the RGD infrastructure and support the RGD commitment to data quality and diversification.

## Supplementary Material

baae132_Supp

## Data Availability

The data referenced in this study are available at https://rgd.mcw.edu. All RGD codes are accessible at https://github.com/rat-genome-database. RGD data, tools, web interface, and code are freely provided to all users, with no requirements for login or license.
